# The Apothecaries’ Act

**Published:** 1869-08

**Authors:** 


### The Apothecaries' Act.

An Act regulating the compounding of prescriptions, has passed both houses of the New York Legislature, and only awaits the Governor's signature to become an operative law. It reads as follows:

Sec. 1. No person employed or in attendance at any drug store or apothecary shop shall prepare a medical prescription, unless he has served two years apprenticeship in a drug store or is a graduate of a medical college or a college of pharmacy, except under the direct supervision of some person possessing some one of the beforementioned qualifications; nor shall any one having permanent charge as proprietor, or otherwise, in any store in which drugs are sold by retail, or at which medical prescriptions are put up for sale or use, permit the putting up or preparation thereof therein by any person, unless such person has served two years as apprentice in a retail drug store, or is a graduate of a medical college or a college of pharmacy.

Sec. 2. Any person violating the provisions of this act shall be deemed guilty of a misdemeanor, and shall be punished by a fine not exceeding $100, or by imprisonment not to exceed six months in the county jail; and in case of death ensuing from such violations, the person offending shall be deemed guilty of a felony, and be punished by a fine not less than $1,000, nor more than $5,000, or by imprisonment in the State Prison for a term of not less than two years or more than four years, or by both fine and imprisonment in the discretion of the Court.

Sec. 3. This act shall take effect immediately.

